# OA25 Not so “savy” afterall

**DOI:** 10.1093/rap/rkae117.025

**Published:** 2024-11-05

**Authors:** Anitha Sokay, Orla Killeen, Emma MacDermott, Kathy Gallagher

**Affiliations:** CHI at Crumlin, Dublin 12, Ireland; CHI at Crumlin, Dublin 12, Ireland; CHI at Crumlin, Dublin 12, Ireland; CHI at Crumlin, Dublin 12, Ireland

## Abstract

**Introduction:**

Interferonopathies (IFN’s) are a constellation of diseases that cause recurrent pathogenic inflammation, arising primarily through antigen-independent hyperactivation of immune pathways¹. It is primarily due to abnormal production or signalling of type I IFNs². A disorder of both the innate and adaptive immune system, this condition is relatively new with the term only coined in 2011. Currently there are more than 20 interferonopathies recognized, with new interferonopathies being identified like Sting associated vasculopathy with onset in infancy (SAVI).

**Case description:**

Our patient first presented with a severe panniculitis rash, Raynaud’s, swelling of her limbs and hepatosplenomegaly at five weeks of age. She developed significant respiratory distress requiring non-invasive ventilation. A CT Thorax revealed diffuse groundglass opacification suggestive of interstitial lung disease. She had elevated inflammatory markers (CRP 24 mg/L, ferritin 1448 ng/ml, ESR 88 mm/hr), a transaminitis (ALT 193 U/ml, AST 356 U/L). She had multiple positive auto antibodies (Table 1) Clinically SAVI was suspected as the most likely diagnosis.

Her interferon signature gene assay demonstrated exaggerated IFI 27 and IFI44L levels. A Trio exome sequencing and IKBKG sequencing however have not been indicative of any currently recognised interferonopathy. Further genetic analysis has been requested to explore possible novel pathogenic mutations. A reasonable clinical response was seen with corticosteroid therapy in combination with Jak Inhibition. With worsening of her cutaneous disease and an increasing need for corticosteroid use a trial of anti IL-6 therapy was initiated. This was subsequently discontinued when she presented with severe acute digital ischemia. It was managed with escalating doses of ruxolitinib, pulse high dose corticosteroids and epoprostenol infusion. Her interferon signature gene assay demonstrated exaggerated IFI 27 and IFI44L levels. A Trio exome sequencing and Inhibitor of nuclear factor kappa B gene (IKBKG) sequencing however have not been indicative of any currently recognised interferonopathy. Further genetic analysis has been requested to explore possible novel pathogenic mutations. A reasonable clinical response was seen with corticosteroid therapy in combination with Jak Inhibition. With worsening of her cutaneous disease and an increasing need for corticosteroid use a trial of anti IL-6 therapy was initiated. This was subsequently discontinued when she presented with severe acute digital ischemia. It was managed with escalating doses of ruxolitinib, pulse high dose corticosteroids and epoprostenol infusion.

**Discussion:**

This case enhances our current understanding of interferonopathies. Although the phenotype and course of the disease is compatible with SAVI³, genetic testing is negative to date. Presently there are eleven heterozygous gain-of-function (GoF) variants for SAVI^4^. With JAK inhibition as the most successful therapeutic option to date^5^. Our case possibly represents an as yet unreported genetic variant, further studies are awaited.This case enhances our current understanding of interferonopathies. Although the phenotype and course of the disease is compatible with SAVI³, genetic testing is negative to date. Presently there are eleven heterozygous gain-of-function (GoF) variants for SAVI^4^. With JAK inhibition as the most successful therapeutic option to date^5^. Our case possibly represents an as yet unreported genetic variant, further studies are awaited.This case enhances our current understanding of interferonopathies. Although the phenotype and course of the disease is compatible with SAVI³, genetic testing is negative to date. Presently there are eleven heterozygous gain-of-function (GoF) variants for SAVI^4^. With JAK inhibition as the most successful therapeutic option to date^5^. Our case possibly represents an as yet unreported genetic variant, further studies are awaited.

**Key learning points:**

• Early suspicion for an interferonopathy in very young infants with interstitial lung disease

• Expanding the potentially new genetic variants of this condition

• Challenge of treatment management

